# Diatoms Green Nanotechnology for Biosilica-Based Drug Delivery Systems

**DOI:** 10.3390/pharmaceutics10040242

**Published:** 2018-11-20

**Authors:** Monica Terracciano, Luca De Stefano, Ilaria Rea

**Affiliations:** 1 Institute for Microelectronics and Microsystems, Via P. Castellino 111, 80131 Naples, Italy; ilaria.rea@na.imm.cnr.it; 2 Materias S.r.l., Corso N. Protopisani 50, 80146 Naples, Italy

**Keywords:** nanotechnology, diatom, biosilica, drug delivery, hybrid devices

## Abstract

Diatom microalgae are the most outstanding natural source of porous silica. The diatom cell is enclosed in a three-dimensional (3-D) ordered nanopatterned silica cell wall, called frustule. The unique properties of the diatom frustule, including high specific surface area, thermal stability, biocompatibility, and tailorable surface chemistry, make diatoms really promising for biomedical applications. Moreover, they are easy to cultivate in an artificial environment and there is a large availability of diatom frustules as fossil material (diatomite) in several areas of the world. For all these reasons, diatoms are an intriguing alternative to synthetic materials for the development of low-cost drug delivery systems. This review article focuses on the possible use of diatom-derived silica as drug carrier systems. The functionalization strategies of diatom micro/nanoparticles for improving their biophysical properties, such as cellular internalization and drug loading/release kinetics, are described. In addition, the realization of hybrid diatom-based devices with advanced properties for theranostics and targeted or augmented drug delivery applications is also discussed.

## 1. Introduction

Drug delivery systems (DDSs) have the capability to amend the limitation of conventional pharmaceutical administration such as poor solubility, short half-life circulation, systemic toxicity, and degradation [1]. The development of new drug molecules is expensive and time-consuming. Hence, the application of nanotechnology to medicine has allowed the development of DDSs able to improve the performance and efficacy of existing pharmaceutic compounds, as well as the quality of life and the longevity of patients [2]. Among all the available nanomaterials for drug delivery applications (e.g., liposomes, dendrimers, polymer, micelles, nanogels, carbon nanotubes, porous silicon (PSi)/silica-, gold- nanoparticles (NPs), etc.), porous silica NPs have been investigated in numerous studies due to their unique properties such as large specific surface area and pore volume, controllable particle size, good biocompatibility, and easy functionalization chemistry [3]. Compared with other synthetic porous silica nanocarriers, mesoporous silica NPs (e.g., MCM-41, SBA-15, M41S) with a pore size ranging from 2 nm to 50 nm turned out to be valid candidates for drug delivery applications [4]. Since their production route is laborious and expensive and involves toxic materials, there is a massive demand to replace these synthetic materials with valid natural surrogates. Surprisingly, nature has provided exciting porous material with three-dimensional (3-D) porous structures: the single-celled photosynthetic diatom algae [5,6,7,8]. Diatom microshells, characterized by unique pill-box micro structures having porosity in the micro/nanoscale range, high surface area, and great biocompatibility, have been shown to be a promising and low-cost biomaterial for drug delivery applications [9,10]. Diatoms, as a source of natural silica, can be generated inexpensively and in enormous amounts through biological algae replication. Diatomite, also known as diatomaceous earth, is a fossil material formed by skeletons of dead diatoms which have accumulated on the bottom of lakes or oceans over millions of years [8,9]. Diatomite is the most abundant source of biosilica and it is largely used as an inexpensive biosilica mineral in several industrial applications (e.g., food industry, agriculture, pharmaceutics, etc.) [5,11]. Despite the great developments in the field of nanotechnology, the diatoms’ architecture can actually compete with man-made fabricated devices [12,13,14]. Due to the high surface area (up to 200 m^2^/g), thermal stability, easy modification through genetic manipulation or chemical modifications, mechanical resistance, optical and photonic properties, non-toxicity, and biocompatibility, diatom frustules are potential scaffolds for the development of nanostructured devices for a variety of applications ranging from liquid filtrations, DNA purifications, immunoprecipitations, photonics, sensing, biosensing, and drug delivery (Table 1) [14,15,16,17,18,19,20,21,22].

This review article presents the recent progress made on diatom biosilica-based system applications for drug delivery applications. The properties of diatom silica used as whole microfrustules or reduced to NPs as non-toxic drug carriers are described. The functionalization of diatom-based micro/nanoparticles that improves their physicochemical properties and drug delivery behavior is also discussed. In addition, the advantage of using hybrid nanodevices obtained by the combination of diatom biosilica and other inorganic NPs (e.g., iron oxide-, gold-NPs, graphene oxide nanosheets) for advanced medical applications is described.

## 2. Diatoms: A Natural Source of Nanostructured Biosilica

**Figure 1:**
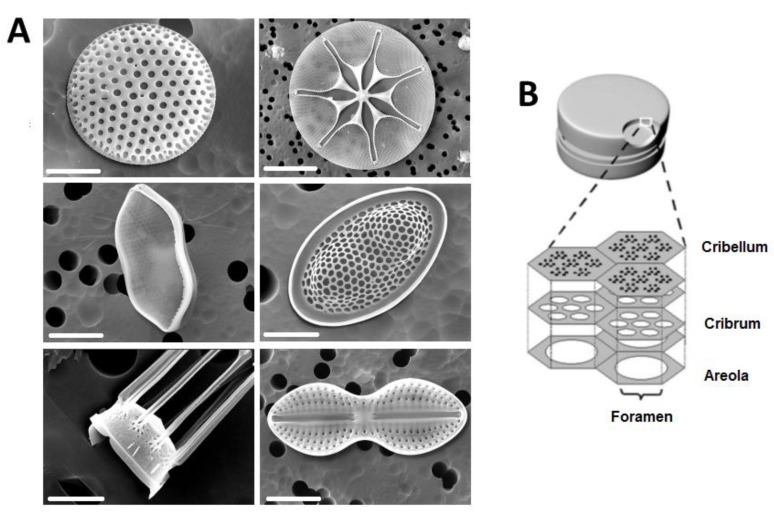
(**A**) Scanning electron microscopy (SEM) images of the cell walls of different diatom species. (**B**) Illustration of a centric diatom frustule with cross-sectional profile of the silica wall typically formed by three overlapping porous layers: cribellum, cribrum, and areola. Reproduced with permission from [28], Springer, 2007.

In recent years, various mesoporous silica particles have been synthesized and applied for drug delivery purposes with the aim to address common therapeutic problems. The production of porous nanomaterials requires bottom-up approaches, such as self-assembling driven chemistry, electrochemical etch, and sol-gel synthesis; and top-down techniques, such as standard or electron beam lithography, nanoprinting, template molding, and atomic layer deposition [18]. All these methods are founded on high-cost equipment and materials, as well as on very skilled personnel, which makes their mass production unattractive from an economic point of view. The advantage of diatoms for nanotechnology over standard methods is that diatoms are easily available, inexpensive, and environmentally friendly material [23]. Diatoms are unicellular photosynthetic algae which colonize every aquatic environment, having an essential impact on the maintenance and development of planet life. Diatom cell walls (called frustules) are the most impressive example of 3-D architectures occurring in nature [24]. There are more than 200 living diatom genera with more than 100,000 estimated species classified by their typical morphologies and size (from 2 µm to 2 mm) (Figure 1A). With respect to frustule symmetry, diatoms are divided into two groups: the centrics are radially symmetrical about an axis that passes through the center of the cell, and the pennates are bipolar symmetrical with the longitudinal axis running parallel to the plane of symmetry [25]. Despite the difference in shape, diatom frustules are typically bipartite structures, with two overlapping valves called thecae. The upper part (epitheca) and the lower part (hypotheca), characterized by a series of linking bands (girdle bands or cingula), are often likened to a Petri dish or pill-box [26,27]. The hierarchical organization of porous elements (e.g., cribellum, cribrum, foramen) contains pore diameters with various patterns ranging from nanometers to micrometers (Figure 1B) [28]. These unicellular algae are the prevalent organisms engaged in the biosilicification process, both in terms of the number of silicified structures they can produce and in the global production of biogenic silica [29]. The biosilicification process occurs in specialized intracellular compartments, named silica deposition vescicles (SDVs). The silicon, collected from the environment in soluble form as orthosilicic acid Si(OH)_4_, penetrates into the cell cytoplasm by specific silicic acid transporter proteins (SITs). These proteins, formed by 10 transmembrane domains, are able to directly bind Si(OH)_4_ via glutamine portions, thus allowing its transport into SDVs. Inside the SDV reaction vessel, Si(OH)_4_ is converted into a silica network (SiO_2_) due to a teamwork of long chain polyamines (LCPAs), silaffin proteins, and polyanionic silacidin peptides [30]. Diatoms can be easily cultivated in large quantities: the cell culture route consists of supplying the inorganic salts as nutrient and sun light for growth [31]. Alternatively, a less expensive source of diatom silica is the diatomite formed by a million years of the fossilization process of dead algae and which is currently mined. Moreover, several studies on the biomineralization mechanism of silica nanofabrication by diatoms have inspired the development synthetic routes to produce novel silica-based materials under mild reaction conditions [32]. Proteins and other organic molecules associated with diatom biosilica have been purified to homogeneity and extensively characterized. In particular, silaniffs and silacidins have been extracted from diatom cell walls and used to successfully synthetize in vitro silica nanostructures such as spheres, roads, or platelets [33].

### Processing of Diatom Frustules for the Preparation of Biosilica Structures

Diatom frustules retain impurities (such as inorganic oxides Al_2_O_3_, Fe_2_O_3_, CaCO_3_, CaO) mainly due the local environment and aging conditions [34]. The treatment of frustules with acid solutions (e.g., sulfuric acid-, hydrochloric acid-based solutions) at different strengths is a valid purification strategy to remove impurities, making them suitable and safe for biomedical applications [35,36]. However, it is fundamental to balance properly the acid solution strength, in order to keep frustule structure integrity. De Stefano et al. described a procedure for cleaning frustules for *Coscinodiscus eailesii*, *Cocconeis*, and *Campyloneis* based on a sulfuric acid solution (97 wt %) for 5 min at 60 °C. The results demonstrated the successful removal of impurities preserving the frustules’ structure [37]. Aw et al. described the purification of diatomite microfrustules with sulfuric acid (1 M) followed by particle size separation using filtration, thus obtaining microcapsules for oral drug delivery. Scanning electron microscopy (SEM), energy-dispersive X-ray spectroscopy (EDXS), and X-ray powder diffraction (XRPD) spectroscopy proved that the obtained structures were composed of amorphous silica and preserved the whole frustule structure [38]. Rea et al. developed a procedure based on crushing, sonication, and filtration of raw diatomite powder, thus obtaining NPs for drug delivery applications. The obtained nanopowder was purified with a piranha solution (2 M H_2_SO_4_, 10% H_2_O_2_, 30 min at 80 °C) and HCL (5 M, overnight at 80 °C). SEM, TEM, and dynamic light scattering (DLS) analysis showed the nanometric size (300 nm) and the porous nature of the obtained powder. Moreover, photoluminescence, Fourier transform infrared (FTIR) spectroscopy and EDXS analysis confirmed the quality improvement of the silica nanopowder after the purification treatment [39]. The diatom biosilica structure after acid/oxidative cleaning can be easily manipulated as a micro- or nano-multifunctional scaffold by various chemical modifications, opening the way to a new class of bioengineered nanostructured materials for biomedical applications [18,38]. The common strategy to develop engineering devices with diatoms is to use the chemistry of silica, which has been highly evolved during the last decades [40]. Frustule surface can be chemically modified by targeting free reactive silanol (SiOH) groups, thus improving drug loading/release properties and adding other reactive groups (−NH_2_, −COOH, −SH, and −CHO), which can be useful for the conjugation of biomolecules (e.g., enzymes, proteins, antibodies, peptides, DNA, aptamers).

## 3. Cytotoxicity Evaluation of Diatom Biosilica Micro- and Nanostructures

**Figure 2:**
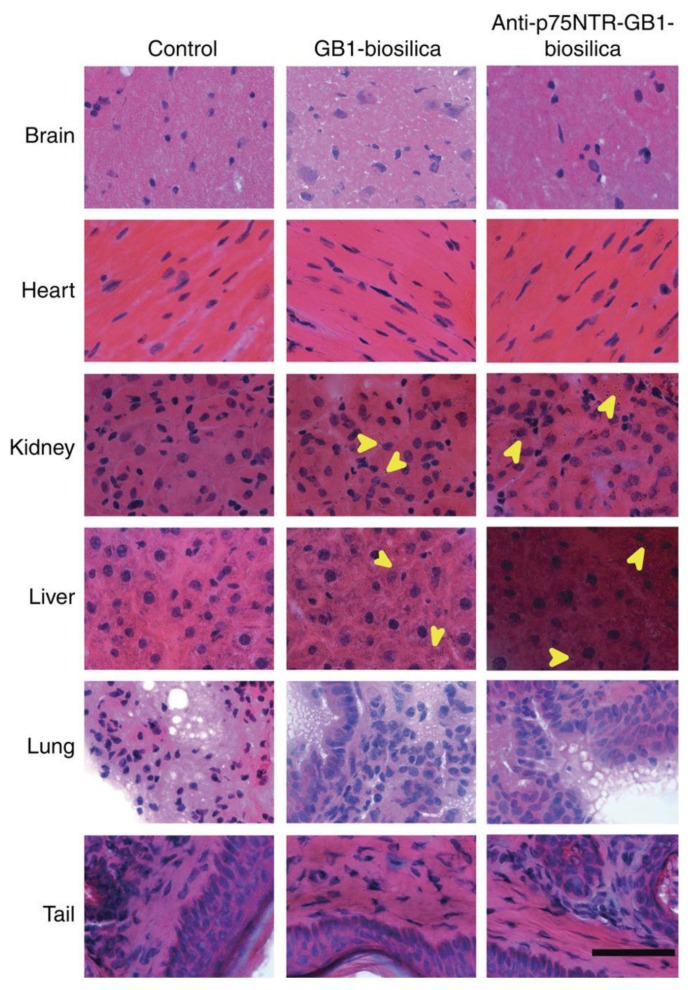
In vivo nude mice biodistribution and tissue damage studies of diatom biosilica structures after eight days of treatment. The tissues appear normal; some biosilica fragments are evident in the kidney and liver samples of mice. Yellow arrows point to biosilica in tissue sections. Scale bar, 50 μm. Reproduced with permission from [43], Nature, 2015.

Biocompatibility and the safe use of new materials in humans are crucial topics in biomaterial science. There are many types of in vitro and in vivo tests that can be used to assess the safety of biomaterials. The in vitro tests, such as cytotoxicity, trombogenity, mutagenicity, and pyrogenity, do not completely determine the safety of materials but are an important step toward animal testing and, finally, clinical trials. The in vivo assessment of biomaterials, by using cavies, is a crucial step to develop and improve all injectable biomaterials for human use. Santos and coworkers described, for the first time, in vitro cytotoxicity in colon cancer cells (Caco-2/HT-29) of diatom microfrustules for oral drug delivery applications [41]. The cell viability assay based on the measurement of adenosine triphosphate (ATP) activity showed low toxicity of diatom microcapsules (up to 1000 μg/mL, for 24 h) against Caco-2/HT-29, confirming the safety of diatoms for drug delivery applications. Rea et al. tested, for the first time, in vitro cytotoxicity of diatomite NPs (approximate size of 300 nm) on epidermoid carcinoma cells (H1355) by 3-(4,5-dimethythiazol-2-yl)-2,5-diphenyl tetrazolium bromide oxidoreductase (MTT) assay. The cells were incubated with different concentrations (20, 100, 200, and 300 μg/mL) of DNPs for 24, 48, and 72 h. The MTT assay showed that H1355 cell viability was not affected even after 72 h of exposure to a diatomite nanopowder concentration up to 300 μg/mL, thus confirming their usability as safe nanovectors in nanomedicine. Actually, a possible limitation of diatomite frustules, in comparison with man-made fabricated porous structures, could be a longer clearance time due to a low dissolution rate at physiological pH [42]. However, this does not seem to be a limitation for diatom biomedical applications. In this context, Voelker et al. conducted in vivo biodistribution studies and assessed whether tissue damage was caused by biosilica diatom structures. After a single intravenous injection into nude mice, the animals were observed daily for eight days and the major organs were then collected. None of the mice exhibited any observable symptoms of acute tissue damage. Optical microscopy studies on tissue sections (8 μm) did not reveal any noticeable abnormality in the major organs, the brain, heart, kidney, liver, and lung, or the tail (Figure 2). Degraded diatom biosilica was observed in liver and kidney sections but not in the lung, and this was confirmed by SEM and EDXS [43].

## 4. Diatom-Based Smart Drug Delivery Systems

Diatoms possess an intricate naturally micro/nanofabricated porous structure with similar physicochemical properties to man-made fabricated porous materials. Characterized by a hierarchical pore structure, large surface area, easily modifiable surface chemistry, good permeability, non-toxicity, high biocompatibility, low cost, and optical and photonic features, diatom frustules have been exploited as alternative cheap scaffolds for the development of innovative devices for drug delivery applications.

### 4.1. Diatom Microcarrier for Oral Drug Delivery

**Figure 3:**
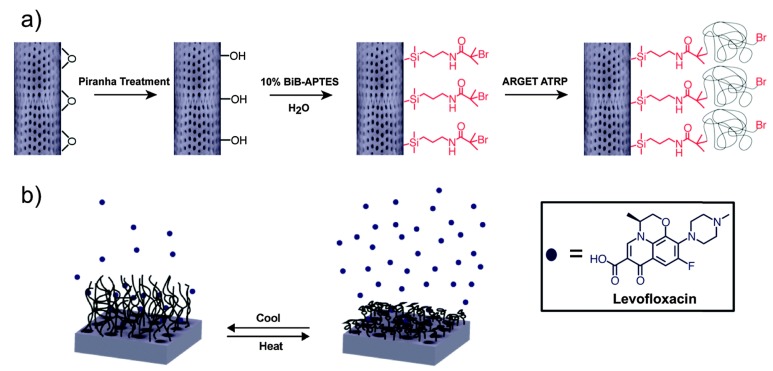
(**a**) Schematic representation of diatom frustule functionalization with thermo-responsive polymer and (**b**) levofloxacin drug release from thermo-responsive polymer-grafted biosilica frustule. Reproduced with permission from [56], Royal Society of Chemistry, 2015.

Over the last decades, the use of diatom frustules in controlled drug delivery applications has greatly increased due to their distinct properties [10,43,44,45,46,47]. Starting from 2010, the first studies on the potential use of bare (i.e., without any surface modification) diatom frustules in drug delivery were based mainly on the encapsulation of therapeutic molecules for oral drug delivery. Losic and co-workers explored the application of diatom microshells for drug delivery of the hydrophobic molecule indomethacin [38]. Results showed the great potential and efficacy of diatom frustules in delivering the drug, with approximately 22 wt % drug loading capability and sustained drug release over two weeks. Principally, two steps of drug release from the frustules were observed: the first over 6 h was rapid due to the surface deposition of the drug, and the second was slow and sustained over two weeks with zero-order kinetics ascribed to the release from the internal hallow structure. Zhang et al. investigated the potential of diatom microparticles for the delivery of mesalamine and prednisone under simulated gastrointestinal conditions, as well as the permeability through Caco-2/HT-29 co-culture monolayers [41]. The results demonstrated the sustained and controlled release of both drugs. The possibility to chemically modify the frustule surface opens the way for improving drug loading/release properties and, moreover, functionalizing their surface with organic-, inorganic-, and bio-molecules, thereby obtaining advanced nanostructured devices. Aw et al. described, for the first time, the impact of organosilane functionalization on diatom silica microcapsules on drug loading and release of water insoluble drug indomethacin [48]. Different compounds (3-aminopropyltriethoxysilane (APTES) and *N*-(3-(trimethoxysilyl)propyl)ethylene diamine (AEAPTMS)) and phosphonic acids (2-carboxyethyl-phosphonic acid and 16-phosphono-hexadecanoic acid) were used to give hydrophilic and hydrophobic features to the frustules. The results showed that an appropriate surface diatom functionalization is able to tune drug loading (15−24 wt %) and release (6−15 days). In particular, the hydrophilic functionalization increased the drug loading and prolonged the drug release, whereas the hydrophobic modification created lower loading and a fast drug release. Milović and co-workers reported, for the first time, the application of diatom as a solid carrier for a water insoluble carbamazepine drug (CBZ) applied in an oral drug delivery system based on the self-emulsifying drug delivery system (SEDDS) [49]. Different solid samples of CBZ suspension in SEDDS were prepared using two methods. The first method was based on the adsorption of CBZ dispersion in SEDDS by a gentle mixing with diatoms; the second method was based on the diatom dispersion in ethanol solution of CBZ/SEDDS components, followed by ethanol evaporation. The dissolution of CBZ from the solid self-emulsifying phospholipid suspension (SSEPS) sample prepared using the second method was faster and better than the sample prepared using the first approach. Higher dissolution was attributed to the loading procedure which was carried out in liquid, and hence a partial adsorption of drug molecules might have occurred inside the pores of the diatoms. Moreover, stability studies under accelerated conditions for 10 weeks demonstrated that diatoms with adsorbed liquid CBZ-loaded SEDDS maintained a polymorphic form of CBZ without a significant influence on the drug dissolution rate and crystallinity, contrary to conventional solid dispersion. Many reports in the literature have demonstrated the great advantages resulting from the use of polymers in the preparation of nanostructured devices for drug delivery applications [50,51,52,53,54,55]. Vasani et al. have demonstrated a controlled drug delivery of antibacterial agent levofloxacin from modified-diatom microcapsules obtained by grafting thermo-responsive oligo(ethylene glycol) methacrylate copolymers on their surface using surface-initiated atom radical polymerization (ATRP) [56]. Drug release experiments from the copolymer modified microcapsules showed strong temperature dependence of the drug release when comparing release kinetics below and above the lower critical solution temperature (LCST) of the grafted copolymer (Figure 3). The antimicrobial action of the released drug was confirmed against two common wound pathogens, proving that diatom frustules can be used as an inexpensive source for the scalable production of drug carriers facilitating controlled therapeutic delivery.

### 4.2. Diatom Nanocarriers for Systemic Drug Delivery

**Figure 4:**
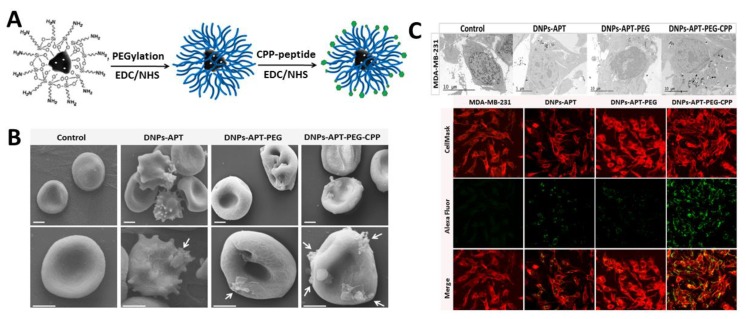
(**A**) Schematic representation of DNP functionalization with PEG and CPP-peptide. (**B**) SEM pictures of the red blood cell morphological modification after the exposure to the modified DNPs. (**C**) Transmission electron microscope (TEM, upper panel) and confocal (lower panel) imaging of MCF-7 cells treated with 50 µg/ml of DNPs-APT, DNPs-APT-PEG, and DNPs-APT-PEG-CPP for 12 h at 37 °C. Adapted with permission from [57], Royal Society of Chemistry, 2015.

**Figure 5:**
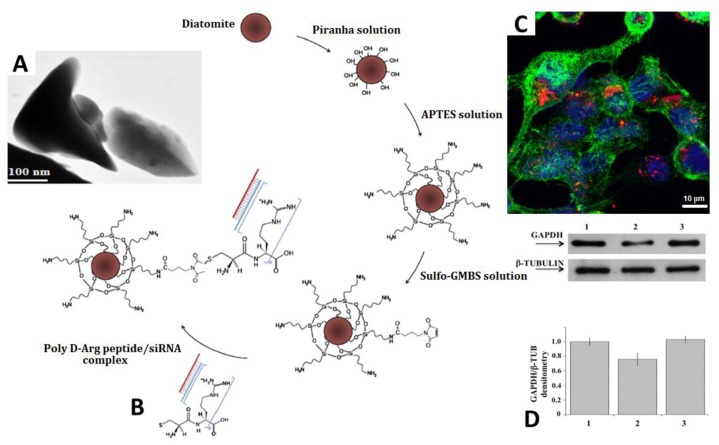
(**A**) Transmission electron microscope (TEM) images of diatomite NPs (DNPs). (**B**) Schematic representation of DNP functionalization with siRNA. (**C**) Representative confocal microscopy image of cells treated with Dy547-labelled siRNA-DNPs for 12 h at 37 °C. Cell nuclei and membranes were stained with Hoechst 33342 and WGA-Alexa Fluor 488, respectively. (**D**) Immunoblotting analysis of GAPDH (upper gel) and of β-tubulin (lower gel) of protein expression in DNPs-siRNA treated cells. Lanes: (1) control cells, (2) DNPs-GAPDH-siRNA, and (3) DNPs-SCR-siRNA. Adapted with permission from [38], Elsevier, 2014.

The development of new effective nanocarriers for targeted drug delivery is expected to solve the problems related to standard therapeutic therapy. In particular, the use of nanocarriers in the treatment of the most common human diseases (e.g., cancer) opens the possibility of directly reaching the desired target side, increasing the drug localization, and reducing the dose of drug used and the systemic side effects. Terracciano et al. reported, for the first time, a study on a biofunctionalization process of diatomite NPs based on PEGylation and cell penetrate peptide bioconjugation (CPP) in order to enhance aqueous stability, improve biocompatibility, reduce cytotoxicity, and increase the solubility of the insoluble anticancer drug sorafenib [57]. Diatomite NPs (DNPs) were obtained through mechanical crushing, sonication of diatom microfrustules, and acid solution purification [17,58]. Subsequently, surface functionalization was carried out with APTES followed by PEGylation via the covalent bond between the carboxyl groups (–COOH) of the PEG chains and the amino groups (–NH_2_) of silanized DNPs using carbamide chemistry. The amino-terminal-PEG-modified DNPs were then conjugated with the carboxyl groups of CPP-peptide, by using the same chemistry (Figure 4). The obtained NPs were stable in aqueous solution and were biocompatible when tested on breast cancer cell lines (MCF-7 and MDA-MB-231) and red blood cells (RBCs). Moreover, the results confirmed that PEGylation and CPP bioconjugation improved the loading/release kinetics of the anticancer drug sorafenib and NPs’ cellular uptake, making them suitable for intracellular drug delivery. Recently, diatom-based nanostructures were explored for gene therapy applications. The first successful attempt to use diatomite NPs for small interfering RNA (siRNA) delivery has been demonstrated by Rea et al. [38]. In this study, siRNA molecules were electrostatically linked to poly-d-arginine peptide covalently bonded to APTES surface-modified DNPs (Figure 5). At first, the safety of the drug-free DNPs (up to 200 μg/mL) was demonstrated after 72 h of incubation with epidermoid carcinoma cells (H1355). The effective delivery of siRNA into cytoplasm with efficient gene silencing was demonstrated. These results suggest that the DNPs as innovative nanocarriers for siRNA transport inside the cancer cells, highlighting the non-toxicity of the material, the efficient cellular uptake, and the gene silencing capability in cancer cells. In a similar context, Martucci et al. described a new personalized B-cell lymphoma therapy based on site-specific receptor-mediated diatomite NPs used as drug delivery systems [59]. Natural silica-based NPs were silanized with APTES and modified with siRNA/poly-d-Arg peptide to actively target antiapoptotic factor B-cell lymphoma/leukemia 2 (Bcl2). The effectiveness of siRNA targeting Bcl2-modified DNPs in downregulation of gene expression was evaluated by quantitative real-time polymerase chain reaction and Western blot analyses. The resulting gene silencing observed was of significant biological importance and opened up new possibilities for the personalized treatment of lymphomas by using DNPs as nanocarriers. Recently, Rea and co-workers investigated the internalization kinetics and intracellular spatial distribution of non-targeting siRNA-loaded DNPs in H1355 lung cancer cells up to 72 h by using label-free Raman spectroscopy [60]. Raman spectra revealed specific bands assigned to DNPs and cellular components, providing evidence that the NPs were internalized and located in endocytic vesicles in the perinuclear region. The analyses demonstrated a considerable NP cellular uptake within 6 h, with equilibrium being achieved after 18 h leading to an efficient distribution of DNPs within the cell cytosol. Moreover, the results showed the presence of DNPs up to 72 h in the cells without damage to their viability or morphology. These data were also confirmed by confocal microscopy and photoluminescence analyses, proving the potential of using non-conventional Raman spectroscopy to study internalization and the fate of NPs in nanomedicine.

## 5. Hybrid Diatom-Based Devices for Biomedical Applications

A major goal in nanomedicine is the implementation of multifunctional platforms within a single targeted nanodelivery system that would simultaneously perform diagnosis, targeted delivery, and efficient therapy [61,62,63,64].

### 5.1. Hybrid Diatom-Based Devices for Drug Delivery

Great efforts have been made in the nanotechnology research field to design systems that integrate multiple components and different materials at the nano scale into a single nanodevice, the so-called hybrid nanodevices [65,66,67]. Fine tuning surface chemical modifications are required to incorporate diatom biosilica with inorganic (graphene oxide, titanium dioxide, etc.), semiconducting (Si-Ge), and metal (Au) scaffolds, thus obtaining hybrid-diatom-based devices [68,69,70,71]. Losic et al. fabricated magnetically guided drug carriers by functionalizing diatoms with dopamine-modified iron-oxide NPs [72]. Dopamine was used to modified iron oxide NPs (DOPA/Fe_3_O_4_) thus forming stable and robust cationic complexes. The magnetized diatoms were obtained by an electrostatically interaction between the cationic magnetic complexes and anionic diatom frustules. The drug release study demonstrated the possibility of obtaining the sustained release of a poorly water soluble drug indomethacin within two weeks, opening the way for magnetically guided target cancer drug delivery by using hybrid diatom silica shells. For the first time, Todd et al. reported iron oxide NPs (IONPs) encapsulated onto diatom frustule (10 μm size) surfaces as magnetically active devices for in vivo delivery of anticancer small molecules [73]. Magnetic resonance and fluorescence imaging were used to investigate the in vivo fate of magnetized diatom frustules. The results demonstrated a significant particle accumulation at tumor site, six times higher than the control, when a magnetic field was applied. This, together with the low toxicity and biodegradability of IONPs and diatom frustules, suggest the great potential of this hybrid nanocomplex for the drug delivery field.

**Figure 6:**
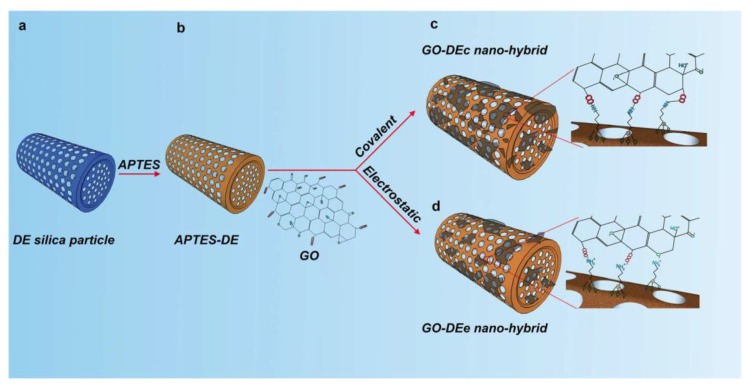
Schematic representation of GO nanosheet functionalization of the diatom frustule surface via electrostatic or covalent chemical approach for the realization of nano-hybrid drug delivery devices. Reproduced with permission from [74], Royal Society of Chemistry, 2013.

Kumeria et al. reported, for the first time, the realization of hybrid microcapsules decorated with two-dimensional (2-D) graphene oxide (GO) nanosheets, thus imparting a pH-dependent triggered release to the systems [74]. Two different approaches for the realization of the nano-hybrids were used: a covalent coupling of GO sheets onto the diatom surface and electrostatic attachment. Diatom structures were silanized using APTES in order to introduce positively amine groups to covalently or electrostatically attach negative GO nanosheets to the frustule surface (Figure 6). The application of the obtained nano-hybrids (GO-DE hybrids) as smart pH-sensitive drug carriers at pH 7.4 and pH 3.5 was demonstrated using a non-steroidal anti-inflammatory drug model indomethacin. The covalently attached GO-DE hybrid devices showed a better drug loading/release capability than electrostatic ones. The results indicated a sustained drug release at pH 3.5 prolonged up to 37 days in comparison to 14 days of the control (APTES-DE). This phenomenon was explained by the enhanced interaction of the drug with GO through H-bonding and hydrophobic interactions to the diatom surface. The release behavior was regulated by the changes in loaded drug and GO nanosheet interactions at different buffer pH.

### 5.2. Hybrid Diatom-Based Devices for Theranostic Applications

**Figure 7:**
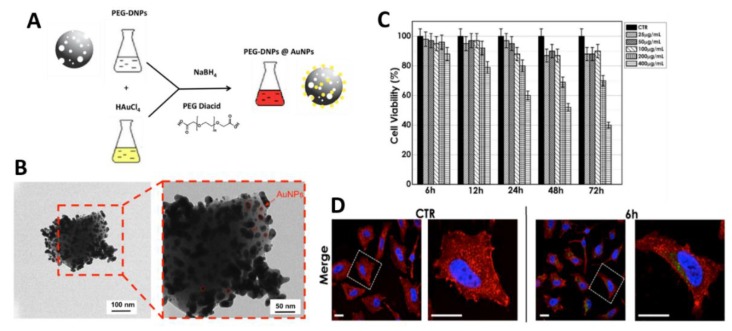
(**A**) Scheme of one-pot liquid phase synthesis method used for decorating diatomite NPs’ surface with gold NPs (PEG-DNPs@AuNPs). (**B**) TEM images of hybrid PEG-DNPs@AuNPs. (**C**) Cell viability of HeLa cells after exposure to 25, 50, 100, 200, and 400 μg/mL of the PEG-DNPs@AuNPs for 6, 12, 24, 48, and 72 h at 37 °C. (**D**) Representative confocal microscopy image of PEG-DNPs@AuNPs (100 μg/mL) labelled with Alexa Fluor^®^ 488 (green) internalized into HeLa cells at 6 h. Cell nuclei and membranes were stained with Hoechst (blue) and WGA-Alexa Fluor^®^ 555 (red), respectively. Adapted with permission from [75], IOPscience, 2018.

Nanosized materials such as magnetic and gold NPs, semiconductor quantum dots, and so on, have been proposed for magnetic resonance imaging (MRI), fluorescent and photoacoustic imaging, contrast agent, drug delivery carrier, cell sorting, and labelling [65,66,67]. Recent literature reported promising results obtained by using hybrid based-porous silica NPs as theranostic nanodevices for both imaging and drug delivery. Recently, Terracciano et al. proposed hybrid gold-diatomite NPs as multifunctional innovative devices for imaging (e.g., photoacoustic, X-ray) and drug delivery purposes [75]. The hybrid complexes (average size of 400 nm) were obtained by decorating polyethylene glycol (PEG)-modified diatomite NPs with gold NPs by a non-conventional one-pot liquid phase synthesis (Figure 7). The inner surface of DNPs was modified by APTES and PEGylated by NH_2_-PEG-COOH in order to improve the DNPs’ aqueous stability and biocompatibility. Moreover, this procedure provided positive amine (–NH_2_) groups on the DNPs’ surface able to adsorb electrostatically the AuCL_4_^−^ ions of gold precursor. PEG-DNPs@AuNPs were obtained by chemical reduction of oxidized Au^3+^ species to metallic Au^0^ particles by using the reducing agent NaBH_4_ in presence of the PEG diacid as a stabilizer. The presence of the PEG diacid onto AuNPs hindered particle aggregation through electrostatic repulsion between its carboxylic groups (–COOH) and the gold surface, conferring a greater stability, as well as biocompatibility, more than conventional stabilizing agents. The formation of PEG-DNPs@AuNPs was macroscopically observed as a color change of the sample solution from pale yellow to deep purple after the addition of NaBH_4_. The nanostructures showed optical properties, displaying a strong surface plasmon resonance band in the UV-vis spectra with peak absorbance at 550 nm. The in vitro cytotoxicity and cellular uptake analysis on human cervix epithelioid carcinoma (HeLa) demonstrated the safety of the nanocomplexes with concentrations up 400 μg/mL for 72 h and an efficient cytoplasmic localization. These preliminary results suggested the suitability of plasmonic PEG-DNPs@AuNPs nanocomplexes as potential theranostic devices in advanced nanomedicine applications.

## 6. Conclusions

Diatoms possess intricate 3-D biosilica porous structures much more advanced and complex than costly man-made fabricated porous materials. Characterized by hierarchical pore structure, great surface area, easily modifiable surface chemistry, good permeability, non-toxicity, and high biocompatibility, diatom frustules have been exploited as a low-cost scaffold for the preparation of innovative devices for drug delivery applications. The present review article outlines the applications of diatom biosilica-based micro-/nanodevices in the drug delivery field. The preparation of diatom micro-/nanocarriers, surface chemical modifications, biocompatibility tests, cellular uptake, drug loading/release capability, as well as targeted therapeutic transport inside cells and advanced drug delivery applications, were discussed. The results presented emphasize the great advantage of using diatom biosilica as an inexpensive alternative to synthetic porous silica for the preparation of the next generation of DDSs.

The progress made in the fabrication of nanoscale diatomite drug delivery systems with additional imaging functionalities, together with the ability to immobilize a targeting ligand on their surface, strongly support the use of these multifunctional devices as nanotheranostic platforms for individualized therapy. Diatomite-based smart nanoparticles should be able to detect diseases (active targeting) and perform submicron visualization (in vivo imaging), therapy, and monitoring of treatment effects in real time. The main advantages could be the early detection of diseases and targeted therapies operating with minimal toxicity.

Even if amorphous silica from diatoms was approved by the Food and Drug Administration (FDA) as Generally Recognized as Safe (GRAS, 21 CFR Section 573.340) for uses in the food industry and agriculture, and classified as not carcinogenic by the International Agency for Research on Cancer, its application in biomedicine has not been yet authorized. Additional studies about the long-term safety of diatomite nanodevices on in vivo animal models are thus required. After this last effort, the hypothesis to concretely commercialize the diatom frustules in the drug delivery field could become practical.

## Figures and Tables

**Table 1:** Properties of diatom frustules and their application in different fields.

Uses	Properties
**Nanotechnology**	Reproducibility of the three-dimensional structures Possibility of genetic engineering and low cost of production Intricate pore sizes which can be modified Ability to self-replicate Natural variability of design includes costae (rib-like structure, further longitudinal rib, and axial rib), canaliculi (tube-like channels), areolae (box-like), punctae (pore-like).
**Biosensing**	Micron-sized and homogenously spaced with striae Optical properties Prospect of cheaply creating thousands of channels on a single silicon chip Low-cost and naturally available material
**Immunoisolation, immunodiagnostics, and immunosensors**	High sensitivity and option to chemically modify the surface to attach bioactive molecules Filtration and encapsulation properties of diatom frustules Evades complements of the immune system
**Filtration and water purification**	Filters micro-organisms Homogeneous permeability and fixed pore size Transport in small numbers Easy multiplication post transport Cost effective United States Environmental Protection Agency (USEPA) approved
**Drug delivery**	Uniform nanoscale pore structure Chemically inert and biocompatible Sustained release of drugs Non-toxic

## References

[1] Cho K., Wang X.U., Nie S., Shin D.M. (2008). Therapeutic nanoparticles for drug delivery in cancer. Clin. Cancer Res..

[2] De Jong W.H., Borm P.J. (2008). Drug delivery and nanoparticles: Applications and hazards. Int. J. Nanomed..

[3] Slowing I.I., Vivero-Escoto J.L., Wu C.W., Lin V.S.Y. (2008). Mesoporous silica nanoparticles as controlled release drug delivery and gene transfection carriers. Adv. Drug Deliv. Rev..

[4] Vallet-Regí M., Balas F., Arcos D. (2007). Mesoporous materials for drug delivery. Angew. Chem. Int. Ed..

[5] Maher S., Kumeria T., Aw M.S., Losic D. (2018). Diatom silica for biomedical applications: Recent progress and advances. Adv. Healthc. Mater..

[6] De Stefano L., Rea I., Rendina I., De Stefano M., Moretti L. (2007). Lensless light focusing with the centric marine diatom *Coscinodiscus walesii*. Opt. Express.

[7] De Stefano L., De Stefano M., De Tommasi E., Rea I., Rendina I. (2011). A natural source of porous biosilica for nanotech applications: The diatoms microalgae. Phys. Status Solidi Curr. Top. Solid State Phys..

[8] Losic D., Mitchell J.G., Voelcker N.H. (2009). Diatomaceous lessons in nanotechnology and advanced materials. Adv. Mater..

[9] Kröger N., Poulsen N. (2008). Diatoms—From Cell Wall Biogenesis to Nanotechnology. Annu. Rev. Genet..

[10] Davidson S., Lamprou D.A., Urquhart A.J., Grant M.H., Patwardhan S.V. (2016). Bioinspired silica offers a novel, green, and biocompatible alternative to traditional drug delivery systems. ACS Biomater. Sci. Eng..

[11] De Medarević D.P., Losic DIbric S.R. (2016). Diatoms–nature materials with great potential for bioapplications. Hem. Ind..

[12] Medlin L.K. (2009). Diatoms (Bacillariophyta). Timetree Life.

[13] Bozarth A., Maier U.G., Zauner S. (2009). Diatoms in biotechnology: Modern tools and applications. Appl. Microbiol. Biotechnol..

[14] Van der Noordaa J., Sol C.J.A., Salimans M.M.M., Jansen C.L., Wertheim-van Dillen P.M.E., van der Noordaa J. (1990). Rapid and simple method for purification of nucleic acids. J. Clin. Microbiol..

[15] Lin X., Tirichine L., Bowler C. (2012). Chromatin immunoprecipitation (ChIP) methodology to investigate histone modifications in two model diatom species. Plant Methods.

[16] De Stefano L., Rendina I., De Stefano M., Bismuto A., Maddalena P. (2005). Marine diatoms as optical chemical sensors. Appl. Phys. Lett..

[17] Ruggiero I., Terracciano M., Martucci N.M., De Stefano L., Migliaccio N., Tatè R., Rendina I., Arcari P., Lamberti A., Rea I. (2014). Diatomite silica nanoparticles for drug delivery. Nanoscale Res. Lett..

[18] Rea I., Terracciano M., De Stefano L. (2017). Synthetic vs Natural: Diatoms Bioderived Porous materials for the next generation of healthcare nanodevices. Adv. Healthc. Mater..

[19] Rea I., Terracciano M., Chandrasekaran S., Voelcker N.H., Dardano P., Martucci N.M., Lamberti A., De Stefano L. (2016). Bioengineered silicon diatoms: Adding photonic features to a nanostructured semiconductive material for biomolecular sensing. Nanoscale Res. Lett..

[20] Terracciano M., Rea I., Stefano L.D., Rendina I., Oliviero G., Nici F., D’Errico S., Piccialli G., Borbone N. (2014). Synthesis of mixed-sequence oligonucleotides on mesoporous silicon: Chemical strategies and material stability. Nanoscale Res. Lett..

[21] De Stefano L., Maddalena P., Moretti L., Rea I., Rendina I., De Tommasi E., De Stefano M. (2009). Nano-biosilica from marine diatoms: A brand new material for photonic applications. Superlattice Microst..

[22] Lettieri S., Setaro A., De Stefano L., De Stefano M., Maddalena P. (2008). The gas-detection properties of light-emitting diatoms. Adv. Funct. Mater..

[23] Gordon R., Losic D., Tiffany M.A., Nagy S.S., Sterrenburg F.A. (2009). The glass menagerie: Diatoms for novel applications in nanotechnology. Trends Biotechnol..

[24] Mann D.G. (1999). The species concept in diatoms. Phycologia.

[25] Guiry M.D. (2012). How many species of algae are there?. J. Phycol..

[26] Hildebrand M., Doktycz M.J., Allison D.P. (2008). Application of AFM in understanding biomineral formation in diatoms. Pflug. Arch. Eur. J. Physiol..

[27] Battarbee R.W., Jones V.J., Flower R.J., Cameron N.G. (2001). Diatoms. Developments in Paleoenvironmental Research.

[28] Losic D., Pillar R.J., Dilger T., Mitchell J.G., Voelcker N.H. (2007). Atomic force microscopy (AFM) characterisation of the porous silica nanostructure of two centric diatoms. J. Porous Mater..

[29] Poulsen N., Sumper M., Kroger N. (2003). Biosilica formation in diatoms: Characterization of native silaffin-2 and its role in silica morphogenesis. Proc. Natl. Acad. Sci. USA.

[30] Kröger N., Lorenz S., Brunner E., Sumper M. (2002). Self-assembly of highly phosphorylated silaffins and their function in biosilica morphogenesis. Science.

[31] Bowler C., De Martino A., Falciatore A. (2010). Diatom cell division in an environmental context. Curr. Opin. Plant Biol..

[32] Kröger N., Rainer D., Manfred S. (1999). Polycationic peptides from diatom biosilica that direct silica nanosphere formation. Science.

[33] Sumper M., Kröger N. (2004). Silica formation in diatoms: The function of long-chain polyamines and silaffins. J. Mater. Chem..

[34] Galal Mors H.E. (2010). Diatomite: Its characterization, modifications and applications. Asian J. Mater. Sci..

[35] Romann J., Chauton M.S., Hanetho S.M., Vebner M., Heldal M., Thaulow C., Vadstein O., Tranell G., Einarsrud M.A. (2016). Diatom frustules as a biomaterial: Effects of chemical treatment on organic material removal and mechanical properties in cleaned frustules from two Coscinodiscus species. J. Porous Mater..

[36] Morley D.W., Leng M.J., Mackay A.W., Sloane H.J., Rioual P., Battarbee R.W. (2004). Cleaning of lake sediment samples for diatom oxygen isotope analysis. J. Paleolimnol..

[37] De Stefano M., De Stefano L. (2005). Nanostructures in diatom frustules: Functional morphology of valvocopulae in *Cocconeidacean monoraphid* taxa. J. Nanosci. Nanotechnol..

[38] Rea I., Martucci N.M., De Stefano L., Ruggiero I., Terracciano M., Dardano P., Lamberti A. (2014). Diatomite biosilica nanocarriers for siRNA transport inside cancer cells. Biochim. Biophys. Acta.

[39] Aw M.S., Simovic S., Addai-Mensah J., Losic D. (2011). Silica microcapsules from diatoms as new carrier for delivery of therapeutics. Nanomedicine.

[40] De Stefano L., Oliviero G., Amato J., Borbone N., Piccialli G., Mayol L., Rendina I., Terracciano M., Rea I. (2013). Aminosilane functionalizations of mesoporous oxidized silicon for oligonucleotide synthesis and detection. J. R. Soc. Interface.

[41] Zhang H., Shahbazi M.A., Mäkilä E.M., da Silva T.H., Reis R.L., Salonen J.J., Hirvonen J.T., Santos H.A. (2013). Diatom silica microparticles for sustained release and permeation enhancement following oral delivery of prednisone and mesalamine. Biomaterials.

[42] Lewin J.C. (1961). The dissolution of silica from diatom walls. Geochim. Cosmochim. Acta.

[43] Delalat B., Sheppard V.C., Ghaemi S.R., Rao S., Prestidge C.A., McPhee G., and Kroger N. (2015). Targeted drug delivery using genetically engineered diatom biosilica. Nat. Commun..

[44] Diab R., Canilho N., Pavel I.A., Ha F.B., Girardon M., Pasc A. (2017). Silica-based systems for oral delivery of drugs, macromolecules and cells. Adv. Colloid Interface Sci..

[45] Ezzati J., Dolatabadi N., Guardia M. (2011). Applications of diatoms and silica nanotechnology in biosensing, drug and gene delivery, and formation of complex metal nanostructures. Trends Anal. Chem..

[46] Uthappa U.T., Brahmkhatri V., Sriram G., Jung H., Yu J., Kurkuri N., Aminabhavi T.M., Altalhi T., Neelgund G.M. (2018). Nature engineered diatom biosilica as drug delivery systems. J. Control. Release.

[47] Terracciano M., De Stefano L., Santos H.A., Lamberti A., Martucci N.M., Shahbazi M.A., Correia A., Ruggiero I., Rendina I., Rea I. Diatomite nanoparticles as potential drug delivery systems. Proceedings of the 2015 International Conference on BioPhotonics (BioPhotonics).

[48] Aw M.S., Bariana M., Yu Y., Addai-Mensah J., Losic D. (2013). Surface-functionalized diatom microcapsules for drug delivery of water-insoluble drugs. J. Biomater. Appl..

[49] Milović M., Simović S., Lošić D., Dashevskiy A., Ibrić S. (2014). Solid self-emulsifying phospholipid suspension (SSEPS) with diatom as a drug carrier. Eur. J. Pharm. Sci..

[50] Hamidi M., Azadi A., Rafiei P. (2008). Hydrogel nanoparticles in drug delivery. Adv. Drug Deliv. Rev..

[51] Liong M., Lu J., Kovochich M., Xia T., Ruehm S.G., Nel A.E., Tamanoi F., Zink J.I. (2008). Multifunctional inorganic nanoparticles for imaging, targeting, and drug delivery. ACS Nano.

[52] Li Z., Barnes J.C., Bosoy A., Stoddart J.F., Zink J.I. (2012). Mesoporous silica nanoparticles in biomedical applications. Chem. Soc. Rev..

[53] Tarn D., Ashley C.E., Xue M., Carnes E.C., Zink J.I., Brinker C.J. (2013). Mesoporous silica nanoparticle nanocarriers: Biofunctionality and biocompatibility. Acc. Chem. Res..

[54] Wang Y., Yan Y., Cui J., Hosta-Rigau L., Heath J.K., Nice E.C., Caruso F. (2010). Encapsulation of water-insoluble drugs in polymer capsules prepared using mesoporous silica templates for intracellular drug delivery. Adv. Mater..

[55] Appel E.A., Tibbitt M.W., Webber M.J., Mattix B.A., Veiseh O., Langer R. (2015). Self-assembled hydrogels utilizing polymer-nanoparticle interactions. Nat. Commun..

[56] Vasani R.B., Losic D., Cavallaro A., Voelcker N.H. (2015). Fabrication of stimulus-responsive diatom biosilica microcapsules for antibiotic drug delivery. J. Mater. Chem. B.

[57] Terracciano M., Shahbazi M.A., Correia A., Rea I., Lamberti A., De Stefano L., Santos H.A. (2015). Surface bioengineering of diatomite based nanovectors for efficient intracellular uptake and drug delivery. Nanoscale.

[58] Terracciano M., Rea I., De Stefano L., Santos H.A. (2018). Chapter 9: Diatoms: A Natural Source of Nanostructured Silica for Drug Delivery. RSC Nanoscience and Nanotechnology.

[59] Martucci N.M., Migliaccio N., Ruggiero I., Albano F., Romano S., Terracciano M., Lamberti A. (2016). Nanoparticle-based strategy for personalized B-cell lymphoma therapy. Int. J. Nanomed..

[60] Managò S., Migliaccio N., Terracciano M., Napolitano M., Martucci N.M., De Stefano L., Rendina I., De Luca A.C., Lamberti A., Rea I. (2018). Internalization kinetics and cytoplasmic localization of functionalized diatomite nanoparticles in cancer cells by Raman imaging. J. Biophotonics.

[61] Razzacki S.Z., Thwar P.K., Yang M., Ugaz V.M., Burns M.A. (2004). Integrated microsystems for controlled drug delivery. Adv. Drug Deliv. Rev..

[62] Xie J., Lee S., Chen X. (2010). Nanoparticle-based theranostic agents. Adv. Drug Deliv. Rev..

[63] Lammers T., Aime S., Hennink W.E., Storm G., Kiessling F. (2011). Theranostic nanomedicine. Acc. Chem. Res..

[64] Rizzo L.Y., Theek B., Storm G., Kiessling F., Lammers T. (2013). Recent progress in nanomedicine: Therapeutic, diagnostic and theranostic applications. Curr. Opin. Biotechnol..

[65] Rosenholm J.M., Meinander A., Peuhu E., Niemi R., Eriksson J.E., Sahlgren C., Linden M. (2009). Targeting of porous hybrid silica nanoparticles to cancer cells. ACS Nano.

[66] Ahmed N., Fessi H., Elaissari A. (2012). Theranostic applications of nanoparticles in cancer. Drug Discov. Today.

[67] Yoo D., Lee J.H., Shin T.H., Cheon J. (2011). Theranostic magnetic nanoparticles. Acc. Chem. Res..

[68] Pytlik N., Kaden J., Finger M., Naumann J., Wanke S., Machill S., Brunner E. (2017). Biological synthesis of gold nanoparticles by the diatom *Stephanopyxis turris* and in vivo SERS analyses. Algal Res..

[69] Chamuah N., Chetia L., Zahan N., Dutta S., Ahmed G.A., Nath P. (2017). A naturally occurring diatom frustule as a SERS substrate for the detection and quantification of chemicals. J. Phys. D Appl. Phys..

[70] Le Q.J., Wang T., Tran D.N.H., Dong F., Zhang Y.X., Losic D. (2017). Morphology-controlled MnO_2_ modified silicon diatoms for high-performance asymmetric supercapacitors. J. Mater. Chem. A.

[71] Lang Y., Del Monte F., Rodriguez B.J., Dockery P., Finn D.P., Pandit A. (2013). Integration of TiO_2_ into the diatom *Thalassiosira weissflogii* during frustule synthesis. Sci. Rep..

[72] Losic D., Yu Y., Aw M.S., Simovic S., Thierry B., Addai-Mensah J. (2010). Surface functionalisation of diatoms with dopamine modified iron-oxide nanoparticles: Toward magnetically guided drug microcarriers with biologically derived morphologies. Chem. Commun..

[73] Todd T., Zhen Z., Tang W., Chen H., Wang G., Chuang Y.J., Deaton K., Pan Z., Xie J. (2014). Iron oxide nanoparticle encapsulated diatoms for magnetic delivery of small molecules to tumors. Nanoscale.

[74] Kumeria T., Bariana M., Altalhi T., Kurkuri M., Gibson C.T., Yang W., Losic D. (2013). Graphene oxide decorated diatom silica particles as new nano-hybrids: Towards smart natural drug microcarriers. J. Mater. Chem. B.

[75] Terracciano M., Napolitano M., De Stefano L., De Luca A.C., Rea I. (2018). Gold decorated porous biosilica nanodevices for advanced medicine. Nanotechnology.

